# Development of symptomatic inflammatory aneurysm treated with endovascular repair in coronavirus disease 2019–infected patient

**DOI:** 10.1016/j.jvscit.2021.03.003

**Published:** 2021-03-23

**Authors:** Christopher J. Riley, Pedro Teixeira

**Affiliations:** Dell Medical School, University of Texas at Austin, Austin, Tex

**Keywords:** Abdominal aortic aneurysm, COVID-19, COVID-19 aneurysm, Endovascular aneurysm repair, Infected aneurysm, Inflammatory aneurysm, Mycotic aneurysm

## Abstract

The sequelae and complications of coronavirus disease 2019 infection have continued to emerge during this pandemic era. Although the long-term effects are continually being monitored and studied, we have been seeing more acute and subacute medical complications requiring a thorough discussion and, sometimes, adjustment of our standard of care and intervention. In the present report, we describe a case of an acute, symptomatic inflammatory or mycotic abdominal aortic aneurysm in a patient with coronavirus disease 2019 who had undergone urgent endovascular repair.

Coronavirus disease 2019 (COVID-19) has rapidly become a pandemic of unexpected proportions. As our knowledge of the virus rapidly progresses, we are learning the sequelae of the disease course. Significant information has been collected and learned regarding the more obvious respiratory and intestinal symptoms associated with the disease. We have been, however, noting an increase in the incidence of inflammatory sequelae affecting multiple organ systems in patients who have acquired and/or recovered from the disease, especially the cardiovascular system.^1^^,^^2^ Mycotic and inflammatory aortic aneurysms represent ~5% to 10% of the total aortic aneurysmal incidence.^3^, ^4^, ^5^ These types are associated with periaortic inflammation not noted in atherosclerotic aneurysm disease.^5^ The patient provided written informed consent for the report of her case.

## Case report

**Fig 1:**
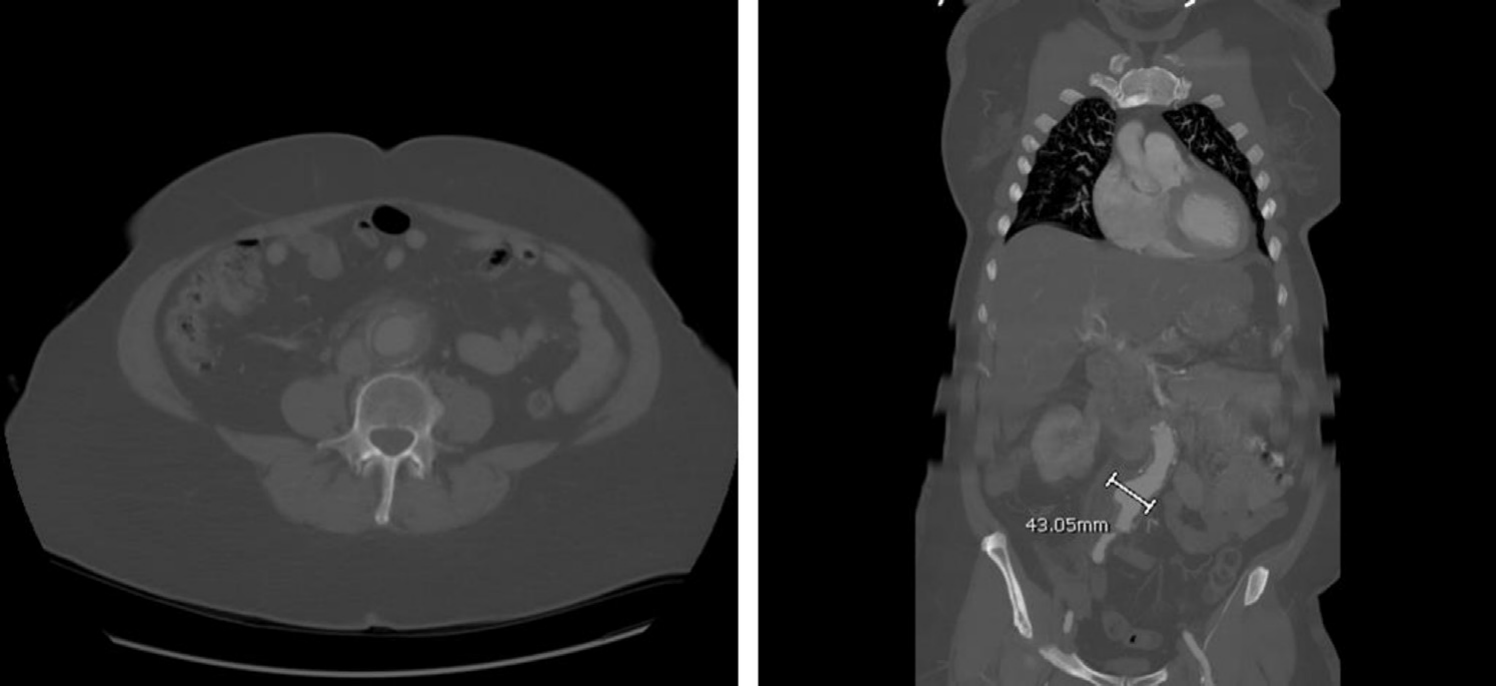
Axial (**Left**) and coronal (**Right**) views of initial computed tomography (CT) scan showing a 4.3-cm aneurysmal segment of the abdominal aorta.

A 57-year-old woman with a medical history significant for coronary artery disease (a history of stent placement), hypertension, smoking, and hyperlipidemia had presented with a 1-day history of worsening abdominal pain. She also reported diarrhea, fever, and a chronic cough that exacerbated her abdominal pain. The initial laboratory test results showed a white blood cell count of 9.4 10^9^/L, C-reactive protein of 4 mg/L, and creatinine of 0.8 mg/dL. An abdominal computed tomography (CT) scan revealed inflammatory changes surrounding an aortic abdominal aneurysm (AAA) measuring 3.8 cm × 4.3 cm (Fig 1). She had no knowledge or history of the aneurysm before her presentation. She was also tested for COVID-19, with a positive test result. Given the presenting symptoms and the acute, inflammatory findings on the CT scan, the differential diagnosis was highly suspicious for an inflammatory or mycotic aneurysm. Blood cultures were obtained, and empiric antibiotics were started. She was admitted for management of her COVID-19 infection, with a vascular surgery consultation for evaluation and management of the AAA. On physical examination, she was moderately tachypneic and diaphoretic, with tenderness to palpation of the left abdomen and flank near the aneurysm region. Her pulses were 2+ palpable in the bilateral upper and lower extremities. During the next 3 days after admission, her abdominal pain progressed. Therefore, a follow-up CT angiogram was obtained. The repeated scan revealed a stable size aneurysm, with, however, increased surrounding inflammatory changes. There were also notably increased infiltrative findings of the right upper lung lobe, consistent with COVID-19 pulmonary manifestations. Given her worsening clinical physical examination, evidence of increasing inflammatory findings, and concern for the risk of rupture in the setting of hemodynamic stability, we decided to proceed with endovascular aneurysm repair (EVAR). Her COVID-19 illness was treated with hydroxychloroquine for 5 days, which was completed 4 days before she underwent EVAR.

**Fig 2:**
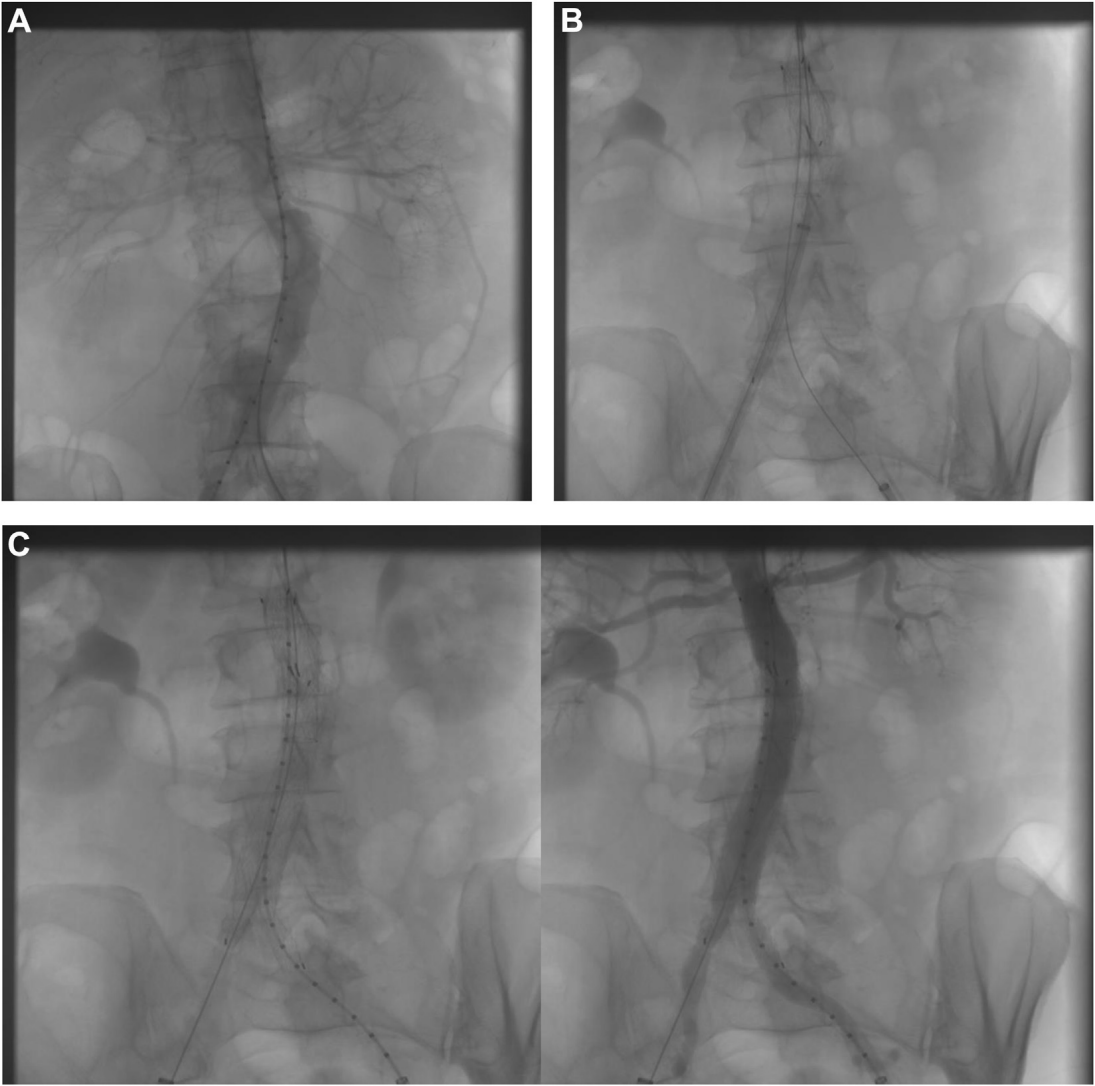
**A,** Angiogram of the abdominal aorta showing the aneurysm. **B,** Fluoroscopic image illustrating deployment of the main body bifurcation stent with extension into the right common iliac artery. **C,** Completion angiogram of abdominal aorta illustrating successful stent placement with exclusion of the aortic aneurysm.

On the day of surgery, the laboratory test results showed a white blood cell count of 10.9 10^9^/L, C-reactive protein of 14.1 mg/L, and creatinine of 1.1 mg/dL. The hospital COVID-19 precautions included the surgical team and staff wearing N-95 masks and full personal protective equipment attire. The procedure was performed in a negative pressure outfitted room with one team inside and one team outside the room boundary to gather additional equipment. Intraoperatively, EVAR was performed in the standard fashion. The initial angiogram (Fig 2, *A*) revealed the targeted region and appropriate size acquired. A 26 mm × 12 mm × 16 cm right-sided endovascular graft was deployed in the infrarenal aorta and right common iliac artery (Fig 2, *B*). After placement of the left common iliac 14-cm excluder, a completion angiogram was performed, which showed satisfactory results (Fig 2, *C*). Postoperatively, she had no hemodynamic instability and experienced immediate resolution of abdominal pain and tenderness. Blood cultures performed on hospital days 1 and 2 continued to show no growth, and the empiric antibiotics were stopped on postoperative day 5. She remained hospitalized for continued management of her COVID-19 pulmonary symptoms and was discharged on postoperative day 5 (hospital day 12).

**Fig 3:**
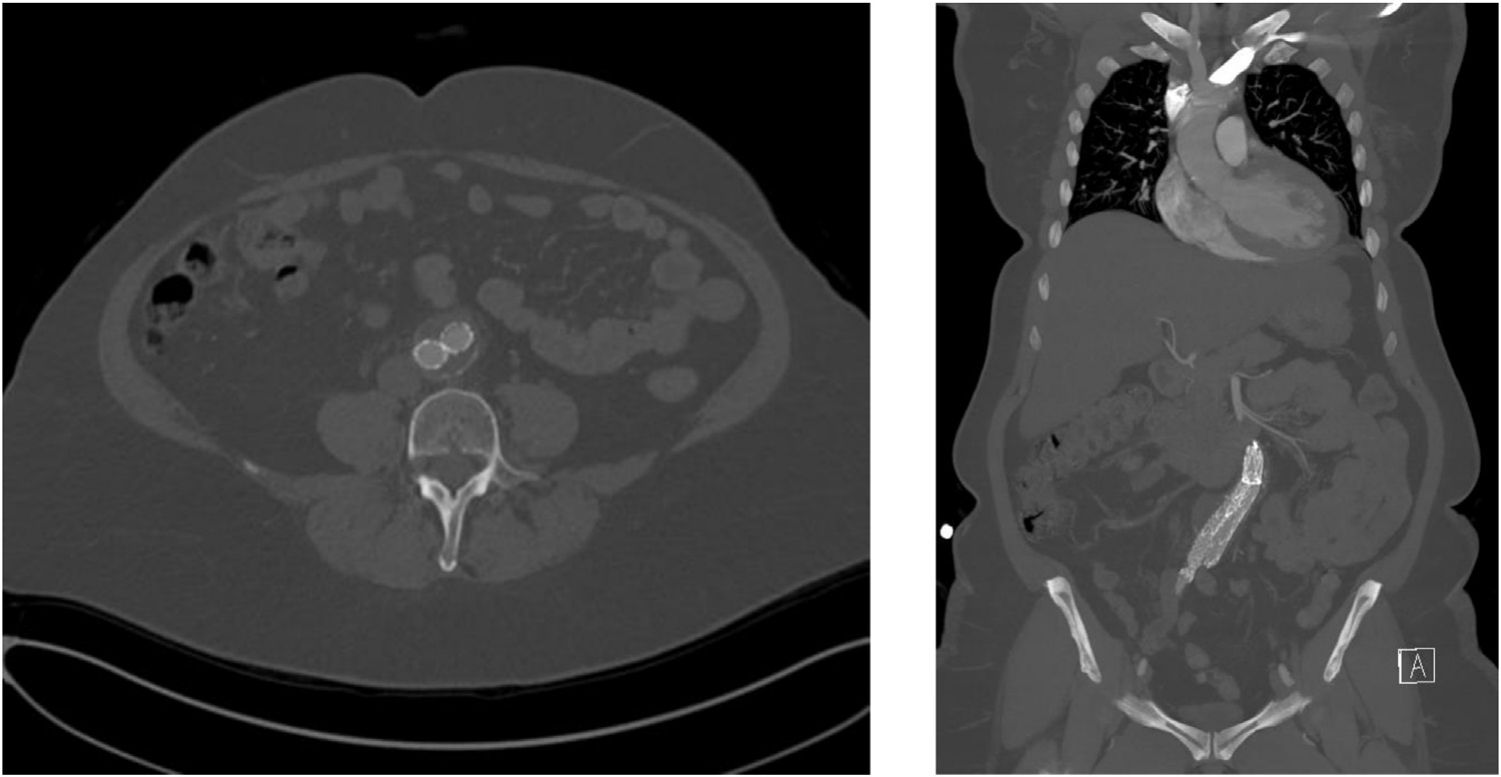
Axial (**Left**) and coronal (**Right**) views of 6-week follow-up computed tomography (CT) scan, illustrating the stent position and continued exclusion of the aneurysm.

A follow-up CT angiogram, performed at 6 weeks after surgery, revealed a successfully excluded aneurysm. The aortic stent was noted to be positioned appropriately, without evidence of an endoleak (Fig 3). The previously noted inflammatory findings had resolved. On examination, she had completely recovered from her COVID-19 pulmonary symptoms and had no abdominal or flank pain.

## Discussion

As the multisystem complications and sequelae resulting from COVID-19 infection continue to be discovered, the approach to evaluating and managing both emergent and nonemergent surgical conditions has evolved.^6^ From a vascular perspective, a significant number of thromboembolic and inflammatory cardiovascular complications have occurred.^2^ The development of aneurysms in the setting of COVID-19 positivity has not been thoroughly explored. EVAR has been increasingly considered a viable first-line option for the repair of AAAs, because the use of the endovascular approach hopes to reduce the risk associated with open dissection in a field of retroperitoneal fibrosis and inflammation.^7^^,^^9^ Although inflammatory aneurysms are less likely to rupture than are the atherosclerotic variant, mycotic aneurysms have a greater risk of rupture, with associated high mortality, given its associated morbidity contributors, such as the development of sepsis.^8^^,^^10^^,^^11^ A high suspicion of a mycotic AAA in the setting of a symptomatic, active, and novel viral infection was our rationale for performing an urgent intervention. Postoperatively, our patient restarted her dual antiplatelet therapy and was prescribed a daily prophylactic dosage of low-molecular-weight heparin. Given the emerging reports and data on the thrombotic complications of COVID-19–infected patients, full anticoagulation therapy with twice daily dosing or even therapeutic dosing could also be rationalized and considered.^12^^,^^13^

The risks and complications of implanting an endograft in a patient with an active COVID-19 infection are unknown. Studies have reported that an endovascular approach is preferable in virally infected patients (eg, human immunodeficiency virus) with operable AAAs.^13^ This approach is, not only thought to decrease the inflammatory response and postoperative complications, but also to decrease the exposure and risk to the operative team.^12^^,^^13^ As COVID-19 transmission continues to be studied, minimizing the risks of exposure to the healthcare teams remains highly important. Reducing the transmission risk, such as from prolonged exposure to intra-abdominal fluids and sharp instruments, support the endovascular approach as a preferable first-line option.

Finally, information and studies regarding an association between a viral illness and aneurysmal development are sparse, even more so for COVID-19. Studies have reported an association between infection with human immunodeficiency virus and the development and progression of vasculopathies, which, in turn, can result in aneurysmal development.^14^ Whether these are truly mycotic in nature has not yet been proved. Evidence has outlined the pathogenetic pathways of COVID-19 and severe acute respiratory syndrome–like viruses and their correlation with the propagation of aortic aneurysmal disease through the upregulation of certain host immune mechanisms and factors by specific viral proteins.^15^^,^^16^ The present case has provided some implications of the possibility of a COVID-19 viral etiology in the development of acute inflammatory and/or mycotic aneurysms.

## Conclusion

The progression of the clinical symptoms and the risk of rupture were the main factors for urgent repair in our patient. Whether the inflammatory etiology was a mechanism of the active COVID-19 infection or resulted from pre-existing factors remains unknown. As we continue to gather reports and data and progressively learn about this novel virus, it is important that we maintain optimal care and the availability of surgical interventions and minimize the risk of complications for patients and the risk of transmission to the operative team. The endovascular approach to inflammatory and mycotic aneurysms is a reasonable first-line option for patients with COVID-19 requiring urgent repair. The long-term risks and sequelae of endograft repair in a patient with an active COVID-19 infection require further study to characterize the risks and benefits. However, in our limited and short-term evaluation, the benefits of the endovascular approach significantly outweighed the risks from a clinical perspective.

## References

[1] Hu Y., Sun J., Dai Z., Deng H., Li X., Huang Q. (2020). Prevalence and severity of coronavirus disease 2019 (COVID-19): a systematic review and meta-analysis. J Clin Virol.

[2] Madjid M., Safavi-Naeini P., Solomon S.D., Vardeny O. (2020). Potential effects of coronaviruses on the cardiovascular system. JAMA Cardiol.

[3] Bisdas T., Teebken O.E. (2011). Mycotic or infected aneurysm? Time to change the term. Eur J Vasc Endovasc Surg.

[4] Hellmann D.B., Grand D.J., Freischlag J.A. (2007). Inflammatory abdominal aortic aneurysm. JAMA.

[5] Ishizaka N., Sohmiya K., Miyamura M., Umeda T., Tsuji M., Katsumata T. (2012). Infected aortic aneurysm and inflammatory aortic aneurysm—in search of an optimal differential diagnosis. J Cardiol.

[6] Verikokos C., Lazaris A.M., Geroulakos G. (2020). Doing the right thing for the right reason when treating ruptured abdominal aortic aneurysms in the COVID-19 era. J Vasc Surg.

[7] Nagahama H., Nakamura K., Matsuyama M., Endou J., Nishimura M., Ishii H. (2013). Inflammatory abdominal aortic aneurysm: report of seven cases. Ann Vasc Dis.

[8] Chaikof E.L., Dalman R.L., Eskandari M.K., Jackson B.M., Lee W.A., Mansour M.A. (2018). The Society for Vascular Surgery practice guidelines on the care of patients with an abdominal aortic aneurysm. J Vasc Surg.

[9] Cardaci M.B., Destraix R., Van Houte B., Vazquez C. (2019). Endovascular repair of inflammatory aortic aneurysms: experience in a single center. Ann Vasc Surg.

[10] Nuellari E., Prifti E., Espositio G., Kapedani E. (2014). Surgical treatment of inflammatory abdominal aortic aneurysms: outcome and predictors analysis. Intervent Med Appl Sci.

[11] Okita Y., Takamoto S., Ando M., Kawashima Y., Matsuo H. (1995). A case report of inflammatory aneurysm of the thoracic aorta. J Vasc Surg.

[12] Rinaldi L.F., Marazzi G., Marone E.M. (2020). Endovascular treatment of a ruptured pararenal abdominal aortic aneurysm in a patient with coronavirus disease-2019: suggestions and case report. Ann Vasc Surg.

[13] Shih M., Swearingen B., Rhee R. (2020). Ruptured abdominal aortic aneurysm treated with endovascular repair in a patient with active COVID-19 infection during the pandemic. Ann Vasc Surg.

[14] Aziz A., Mooka B., Clarke Moloney M., Kavanagh E. (2013). Endovascular management of ruptured common iliac mycotic aneurysm in an HIV-positive patient. BMJ Case Rep.

[15] Nain Z., Rana H.K., Liò P., Islam S.M.S., Summers M.A., Moni M.A. (2020). Pathogenetic profiling of COVID-19 and SARS-like viruses. Briefings Bioinformat.

[16] Cai B., Yang B., Huang D., Wang D., Tian J., Chen F. (2020). STAT3-induced up-regulation of lncRNA NEAT1 as a ceRNA facilitates abdominal aortic aneurysm formation by elevating TULP3. Biosci Rep.

